# Dimensionality Reduction in Lingual Articulation of Vowels: Evidence From Lax Vowels in Northern Anglo-English

**DOI:** 10.1177/00238309251320581

**Published:** 2025-03-25

**Authors:** Patrycja Strycharczuk, Sam Kirkham, Emily Gorman, Takayuki Nagamine

**Affiliations:** Linguistics and English Language, The University of Manchester, UK; Linguistics and English Language, Lancaster University, UK; Speech, Hearing and Phonetics Sciences, University College London, UK

**Keywords:** Articulation, vowels, ultrasound

## Abstract

There is a long-standing debate on the relevant articulatory dimensions for describing vowel production. In the absence of a theoretical or methodological consensus, different articulatory studies of vowels rely on different measures, which leads to lack of comparability between different sets of results. This paper addresses the problem of how to parametrise the tongue measurements relevant to vowels, obtained from midsagittal articulatory imaging. We focus on the lax vowels subsystem in Northern Anglo-English. A range of measures quantifying tongue position, height, and shape are extracted from an ultrasound dataset representing 40 speakers. These measures are compared, based on how well they capture the lingual contrast between different vowels, how stable they are across different speakers, and how intercorrelated they are. The results suggest that different measures are preferred for different vowels, which supports a multi-dimensional approach in quantifying vowel articulation.

## 1 Introduction

Dimensionality reduction is an important step in quantitative phonetic analysis, connecting issues in phonetic theory, methodological practice, and interpretability of findings. We use the term “dimension” as representing a possible physical measurement, and by “dimensionality reduction,” we understand the selection of a small set of key representative measurements. There are areas where the theory does not deliver a single clear methodological pathway for dimensionality reduction, with consequences for our understanding and replicability of phonetic research. In this paper, we consider which dimensions are preferred for representing articulatory information in vowel sounds, and specifically information related to the tongue. We first argue that different theoretical ideas suggest different practical approaches to this problem. We then proceed to make a systematic comparison of different possible methodologies, and based on this evaluation, we make methodological recommendations situated in the context of theoretical frameworks for articulatory representation of vowels.

For some articulatory imaging techniques, such as electromagnetic articulography, a degree of dimensionality reduction is integral to the method, as articulator movement is reduced to the movement of fleshpoints. Others, like ultrasound tongue imaging, provide rich holistic information about the overall shape and movement of the tongue which needs to be reduced if quantitative comparisons are to be made. Such comparisons may concern differences between groups of speakers, or between experimental conditions. As an example, let us consider an ultrasound study that wants to establish lingual differences underpinning different degrees of ongoing vowel fronting in different groups of speakers. What is the best way to quantify the relevant information about the tongue for such a comparison? Ideally, the answer to this question would be informed by a theoretical model of speech production. However, there are multiple such models, and an associated multitude of views on the relevant dimensions in the articulation of vowels, as we discuss below.

### 1.1 Theoretical models of vowel production

One of the most established and familiar models of vowel production is the tongue-arching model, widely attributed to Bell (1865), and popularized by Jones (1922), who used it as the basis of the cardinal vowel system, adopted as a reference frame by the International Phonetic Association. The model assumes that there are two degrees of freedom for tongue movement in vowel production: high-low and front-back, with lip rounding as the third dimension. Four cardinal vowels, /i, a, ɑ, u/, are defined by Jones as articulatory extremes representing high-front, low-front, low-back, and high-back corners of the vowel space. In addition, Jones specified the tongue position and height in terms of position and height of the relatively highest point on the tongue. While a prominent aspect of the model is articulatory, the cardinal vowels model also has an auditory component, and it was originally intended as a framework for auditory transcription and phonetic teaching. In this, the model has been tremendously successful and influential. It has also been a lasting influence on articulatory interpretation of auditory/acoustic relationships between vowels, even though the articulatory accuracy of the model has been called into question since the early days after its inception.

Ever since tongue imaging evidence started accumulating, it became clear that the schematised relationships between vowels, as captured by the vowel quadrilateral, do not closely match the articulatory reality. Observations of these issues go back to the work by Russell (1936) and include considerable height asymmetries between front and back vowels (Ladefoged & Maddieson, 1996, 284), and the so-called vowel-flips, that is, cases when auditory height relationships are reversed in acoustics, for example, the highest point for /e/ is higher than the highest point for /ɪ/ (Johnson et al., 1993; Noiray et al., 2014; Wood, 1982). Staunch critics of the model have included Ladefoged (1971, 67–80) and Wood (1982). Other evaluations of the tongue-arching model have been more balanced (Catford, 1981; Fischer-Jørgensen, 1984; Lindau, 1978), but they nevertheless acknowledge some shortcomings and discuss the model in terms of a broader articulatory abstraction rather than a precise framework that captures the tongue articulation.

Some alternative proposals to Jones’s original model embrace the notions of tongue position and tongue height but suggest different measures compared to the coordinates of the highest point on the tongue. For example, Fischer-Jørgensen (1984) redefines tongue position as the position of the tongue body, and tongue height as the distance from the constriction articulator to the palate. Ladefoged and Maddieson (1996) propose to use the position of the highest point on tongue relative to front incisors as a measure of tongue position, and the distance between the highest point on the tongue and the roof of the mouth as a measure of height. Catford (1977) develops a two-dimensional model based on the polar, as opposed to the Cartesian, coordinate system, with /a/ serving as the origin, and equidistant radials linking /a/ with /i/, /ɨ/, /o/, /ɔ/, and /ɑ/. In doing so, the model captures some inherent correlations between position and height.

Other two-dimensional models propose a more radical departure from the tongue position and tongue height, however defined. The acoustic theory of speech production redefines the two key dimensions relevant to vowel production in terms of the volume of the resonating cavities within the vocal tract (Fant, 1971; Stevens & House, 1955). Stevens and House (1955) argue that while the speech targets are acoustic (speakers target specific resonances), most of the target resonance is controlled by the tongue. They propose that the tongue has two degrees of freedom: location of lingual constriction and degree of lingual constriction. They provide a formula for these dimensions based on the distance of the constriction from the glottis and the radius of the tube at the point of constriction. The second measurement is related to the size of the region in a two-dimensional midsagittal view of the vocal tract. The fundamental notions of inter-articulator distance and area size also underlie the research tradition of estimating area functions in imaging of the vocal tract (Fant, 1971; Perkell, 1969; Perrier et al., 1992; Story et al., 1996).

Maeda (1990) proposes a two-dimensional model of tongue displacement in vowels, based on a theoretically constrained but data-driven dimensionality reduction of cineradiographic data of vocal tract movement. He identifies the two lingual components to be tongue position and tongue shape. Honda (1996) provides a muscle-based interpretation of these components as the products of actions of two pairs of functionally antagonistic muscles. Tongue position is related to the actions of posterior genioglossus and hyoglossus, which generate tongue advancement and raising as well as tongue lowering and retraction, respectively. The other dimension, tongue shape, is related to the activity of styloglossus and anterior genioglossus, which respectively control the backwards and up vs. downward and front movement (note that Honda et al. (2010) provide a later re-interpretation of the role of the styloglossus).

The aforementioned examples demonstrate that a common thread runs through multiple models of vowel production, identifying two major components at the level of muscle control, tongue configuration, resonant cavity volume, acoustic signal, and auditory perception. The parallels between these models grow out of broad regularities concerning vowel contrasts that can be observed across different levels and underlie the correspondence between multiple processes within the speech production-perception loop. However, there is also an alternative theoretical lineage that proposes modeling lingual articulation using a greater number of dimensions. We will refer to this general approach as multi-dimensional (as opposed to two-dimensional) models.

According to Wood (1982), the idea of multi-dimensional nature of lingual vowel articulation is very old, going all the way back to ancient Indian grammarians, who proposed that there are three primary locations of vowel constriction: palatal, labial-velar, and pharyngeal. Wood (1979, 1982) expands this model to differentiate between two pharyngeal constriction regions: upper and lower. The dimensions are fundamentally consistent with muscle actions, as proposed by Honda (1996). Posterior genioglossus is active in generating palatal vowels /i, ε/; styloglossus in velar vowels /u, ʊ/; anterior genioglossus in lower pharyngeal vowels /a, ɒ/; and hyoglossus in upper pharyngeal vowels /o, ɔ/. According to Wood (1979, 1982), the basic taxonomy of four constriction locations is also in line with observations concerning the quantal nature of vowels, as these constrictions represent areas where the acoustic output is robust to articulatory variability (Stevens, 1989). Thus, Wood’s model is at some level compatible with some two-dimensional approaches. Crucially, however, quantifying the four constriction locations within the model itself is not two-dimensional, as the constriction is specified separately for the different relevant articulators.

Rich multi-dimensional specification is also a feature of the Articulatory Phonology/Task Dynamic model (Browman & Goldstein, 1986, 1992), which represents speech production in terms of time-varying displacement of multiple articulators. Within this model, multiple parts of the tongue are specified separately, including the tongue root, tongue body, and the tongue tip. While not all individual articulators would be considered to be under active control for the production of any particular target, a complete specification would extend to several parts of the tongue, as well as other articulators.

Another multi-dimensional model is advanced by Esling (2005), who reconceptualizes the articulatory vowel space as a product of two articulators: lingual and laryngeal. The lingual articulator has three parameters, representing front, raised, and retracted vowels, defined in accordance with primary muscle groups, as identified by Honda (1996). These tongue muscle actions interact with the movement of the laryngeal articulator, creating two resonance cavities, one of which is primarily lingual, and the other primarily laryngeal.

In summary, theoretical models of vowel production diverge in their views of how many dimensions there are in articulatory vowel production, and how they relate to the degrees of freedom of tongue movement. Accordingly, we also find a multitude of practical approaches to representing tongue-related information in articulatory vowel studies, as discussed in Section 1.2.

### 1.2 Methodological practice in lingual dimensionality reduction

Some articulatory studies of vowels take a data-driven approach to characterizing tongue movement. This typically involves using parameters that emerge from statistical dimensionality-reduction techniques, such as principal component analysis or factor analysis (Carignan et al., 2015; Harrington et al., 2011; Harshman et al., 1977; Hoole et al., 2016; Johnson et al., 1993; Mielke et al., 2017; Stone et al., 1997; Story & Titze, 1998; Strycharczuk et al., 2021). Such techniques use the coordinates representing multiple points on the tongue surface as the input, and the multi-dimensional data are subsequently used to identify two or three components, depending on the threshold of cumulative variance explained. The components can be interpreted post hoc to some degree, although this may not always be possible, depending on the nature of the data.

Because of the interpretability issues, it may sometimes be more appropriate to predetermine the relevant phonetic dimensions along which differences are measured. A survey of the literature suggests that many studies that do so rely on two dimensions, but there is variation in how these dimensions are defined. Several ultrasound studies use the coordinates of the highest point on the tongue to capture the tongue position and/or height (Albuquerque et al., 2022; Dokovova et al., 2019; Georgeton et al., 2016; Hussain & Mielke, 2021; Lawson et al., 2019; Ménard et al., 2012; Noiray et al., 2014; Song, 2017). Across these studies, frontness and backness are operationalized in different ways. Some use the occlusal plane to standardize the rotation (Albuquerque et al., 2022; Dokovova et al., 2019; Hussain & Mielke, 2021; Noiray et al., 2014). Scobbie et al. (2012) note that occlusal plane is only one of the possible approaches to standardization in this context and compare it to another approach, which uses a tangent between /i/ and /o/ to compare vowels, where the point of measurement is defined as the smallest difference from the tongue contour for each vowel to the /i/-/o/ tangent. The two approaches are similar in capturing some of the relative vowel distances, but they inevitably differ in finer detail concerning vowel distance. Ménard et al. (2012) propose yet another approach, defining the highest point as the point on the tongue surface that is relatively farthest away from the line connecting the most anterior and most posterior points on the tongue contour.

Apart from the coordinates of the highest point on tongue, several other measures of front-back and high-low dimensions of tongue displacement are used in ultrasound experimentation. Kirkham and Nance (2017) quantify position in terms of position (X-coordinate) of the tongue root and use the height of the highest point of tongue (i.e., maximum tongue raising) as the measure of tongue height. Strycharczuk and Scobbie (2017) use the X-coordinate at which the posterior part of the tongue crosses the occlusal plane as the measure of position. Hussain and Mielke (2021) define tongue dorsum position as the angle in radians from the origin to the most distant point of the tongue trace. In addition, they quantify the position of the tongue root as the X-coordinate of the posterior edge of the tongue trace (based on occlusal rotation). Havenhill (2024) defines tongue position as the X-coordinate of the posterior part of the tongue dorsum. Lee et al. (2015) develop a measure of tongue height that involves distance from the tongue dorsum to the plane of mylo-hyoid muscle. The other articulatory dimension they propose is termed “tongue aperture,” but it could be considered a measure of tongue position, as it is orthogonal to height and uses the same landmark (tongue dorsum) as the reference point.

We can generalize that the measures of high-low and front-back tend to be taken using the highest point on the tongue as the landmark, or using an anatomically-defined reference point, such as tongue root or tongue dorsum. Similar variation is found in the methodologies of EMA (electromagnetic articulography) studies of vowels. For example, Iskarous (2010) relies on the X-Y coordinates of the point on the tongue surface that is closest to the palate. This landmark is intended to represent the constriction point, as is conceptually similar, although not identical to the highest point on the tongue. In contrast, Blackwood Ximenes et al. (2017) rely on an anatomically defined landmark, visualizing articulatory vowel spaces in terms of X-Y coordinates of the sensor placed on the tongue dorsum. Similarly, Gorman and Kirkham (2020) use the X-position of the tongue dorsum sensor as a measure of vowel fronting.

Instead of the high-low dimension, some articulatory studies focus more specifically on the tongue shape. Several measures have been proposed in the literature to capture the shape complexity. Ménard et al. (2012) develop curvature measures based on a triangle fitted between the edges of the visible tongue contour and the point on the tongue surface farthest removed from the base of the triangle. Stolar and Gick (2013) propose a lingual curvature index, which is an integrated measure of curvature, based on a polynomial fit to the tongue surface. The measure captures the complexity of the tongue shape, corresponding to differences in manner of articulation. Liquids, which are frequently characterized by the presence of multiple constrictions, show higher complexity than other consonants, and consonants show higher complexity than vowels. Dawson et al. (2016) modify this measure by calculating the absolute curvature of the tongue at equally spaced points on the tongue surface and integrating it. They show that the measure can differentiate between a range of tongue shapes, including some vowel contrasts. Hussain and Mielke (2021) propose a measure of tongue dorsum concavity and tongue blade angle to capture shape contrasts specific to rhotic vowels.

To sum up, experimental studies of lingual articulation in vowels differ considerably in the parameters they use, and also in the way these parameters are operationalized. The methodological discrepancies mirror the wide range of theoretical perspectives on the nature of tongue articulation in the production of vowels. While the links between theory and practice are not always explicitly acknowledged, we can reconstruct some of them. The use of the highest point on the tongue is rooted in Jones’s (1922) tongue-arching model. Measuring the displacement of particular anatomical points (e.g., tongue root, tongue dorsum) is more typical of a modified tongue-arching model, in which high-low and front-back dimensions are redefined. Alternatively, this approach may also represent displacement of individual articulators, as in multiple articulator models (such as those of Browman & Goldstein, 1986, 1992). Methodologies that rely on inter-articulator distance are consistent with resonance-based theories, as originally proposed by Stevens and House (1955). Methods for quantifying tongue shape are conceptually close to muscle-based models, such as Honda (1996).

An important question that emerges in this context is how the different methodologies compare. Are they conceptually different but practically equivalent approaches, or do they carry with them systematic differences that also have consequences for the type of information we extract, and ultimately for our empirical understanding? The present study sets out to tackle this problem by comparing the quality and the content of the information delivered by selected articulatory measures of vowels.

### 1.3 This study

In this study, we aim to probe deeper into the nature of different articulatory measures of vowels, the interpretation of these measures, and the relationships between them. We address the question of how well different measures of tongue position and height/shape capture global 2-D midsagittal tongue surface variation between lax vowels in Northern (Anglo-) English, imaged using ultrasound. While we work with ultrasound data, we believe that the findings can be generalized to other methods of tongue imaging, such as EMA or MRI.

We chose to study lax vowels for several reasons. They represent a small, reasonably symmetrical and phonologically coherent subsystem. For our purposes, it is helpful to work with a relatively small vowel set because we want to be able to compare different measures for specific vowel contrasts, and reducing the set of possible contrasts makes this process more manageable. Furthermore, lax vowels are unquestionably monophthongal in Northern English, and they can therefore be reasonably reduced to a single target. This property of lax monophthongs provides the theoretical rationale for using static representations of vowels, corresponding to a single time point per each vowel token. In contrast, a single-target representation may be less appropriate for long vowels, as we have argued in the article by Strycharczuk et al. (2024). With all that said, we are conscious that focusing on lax vowels introduces its own challenges, insofar as these vowels are articulatorily and acoustically less distinct, compared to tense vowels in English. We return to this issue in Section 4.

We evaluate a range of measures of tongue position, tongue height, and tongue shape. Tongue position (degree of frontness/backness) is widely considered as the main locus of variance for lingual articulation of vowels. Depending on the model, the secondary measure is commonly taken to be either tongue height or tongue shape, based on the survey of experimental literature presented in Section 1.2. The specific measures we examine are described in Section 2.5.1.

**Figure 1.**
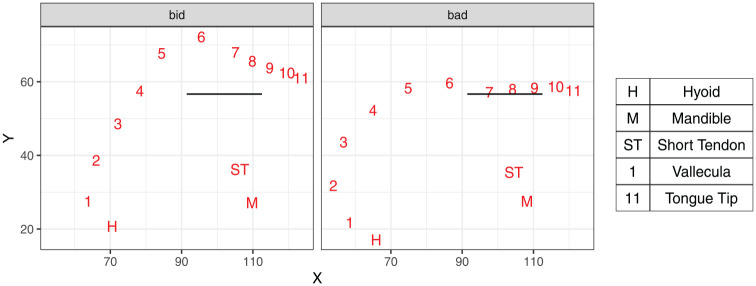
DLC landmarks as identified for an example token of *bid* and *bad* by speaker LNCS2. The horizontal line corresponds to the occlusal plane.

Our analysis builds on a recent methodological development involving application of the DeepLabCut algorithm (DLC, Mathis et al., 2018) to identifying consistent landmarks on the tongue contour. Wrench and Balch-Tomes (2022) present a validation of this application for tongue data. The landmarks are 11 points (knots) on the tongue surface, as shown in Figure 1 for two example tokens produced by the same speaker. The most posterior knot (Knot 1) corresponds to the vallecula, and the most anterior knot (Knot 11) is placed on the tongue tip. Nine DLC knots are spaced at equal distances along the tongue surface, with two additional knots present in the tip/blade area to capture the wider range of articulatory contrasts affecting the anterior part of the tongue. We can generalize that knots 2 and 3 correspond to the tongue root, whereas knots 5–7 are on the tongue dorsum. In addition to the 11 knots, DLC identifies the hyoid, the mandible, and the short tendon attached to the mandible.

We investigate whether the X-Y coordinates of different knots can serve as a proxy for the overall midsagittal tongue height and position and what differences follow from selecting different knots for this purpose. We further compare the anatomically-defined tongue coordinates with the X-Y coordinates of the highest point on the tongue (defined using different reference points) and with measures of tongue curvature (TC).

The research questions guiding the comparisons between measures are as follows.

1. How well do different measures capture the overall midsagittal lingual contrast between vowels
2. How consistent are these measures across speakers?
3. What information about vowel contrasts is replicated across different measures, and what information is unique to specific measures?

## 2 Methods

### 2.1 Stimuli

**Figure 2.**
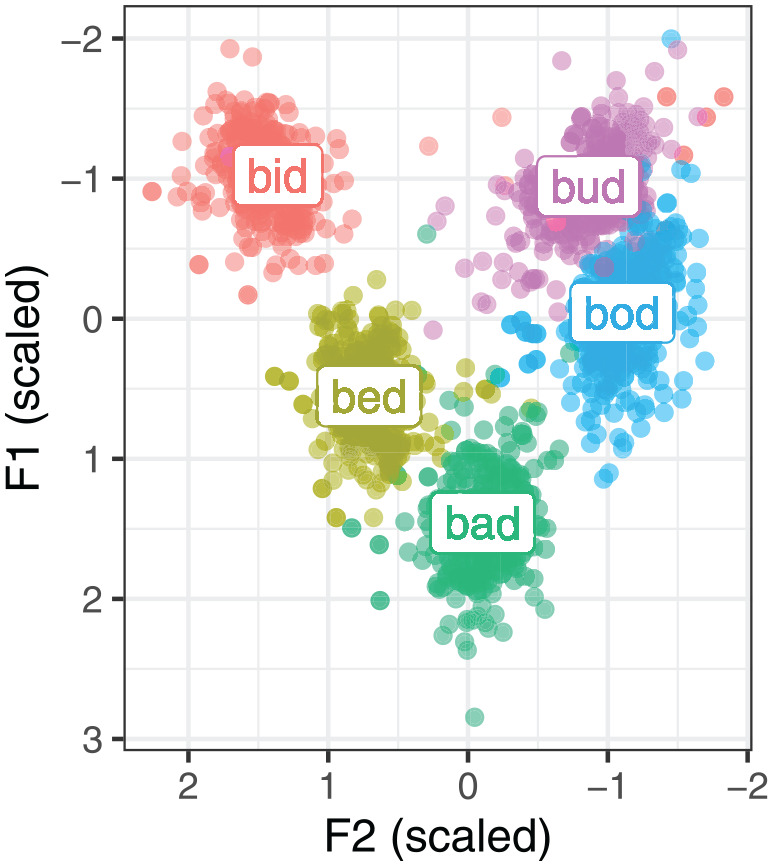
Normalized (scaled) Hertz measurements of the stimuli vowels taken at the acoustic midpoint.

The stimuli used in this study represent the five lax vowels of Northern English, kit, dress, trap, lot and strut, embedded in a b _ d segmental context (*bid, bed, bad, bod, bud*). The use of a stable segmental context avoids segmental confounds and ensures that the differences we observe about configuration for different vowels are due to the vowel itself. Note that strut and foot lexical sets are merged for most present-day speakers of Northern English, or rather, the two sets have not undergone the historical split present in most other varieties of English (Baranowski & Turton, 2018; Strycharczuk et al., 2019; Wells, 1982). The acoustic quality of the five vowels is shown in Figure 2. The stimuli were selected from a larger corpus comprising a wider set of vowels. The stimuli were embedded into two types of carrier phrases: *She says X*, and *She says X eagerly*.

### 2.2 Speakers

Forty native speakers of Northern Anglo-English participated in the recording. Twenty-eight speakers were from Greater Manchester, 10 were from Lancashire, and two from other locations in the North of England (Cumbria and Yorkshire). The age range was 18–48 (mean = 24.6). Twenty-three participants described their gender as female, 15 as male, one as non-binary, and one did not answer the gender question. Thirty-four speakers described their ethnicity as White, three as Asian British, one as Mixed White and Asian, one as Other (these categories correspond to the ones adopted by the UK Census), and one did not answer the ethnicity question. All the participants declared no history of speech or hearing disorders. They all signed a consent form and received a small compensation for taking part.

### 2.3 Procedure

The data were recorded in a laboratory setting across two sites. The recordings took place in 2022 and 2023. Thirty-one speakers were recorded at the University of Manchester Phonetics Lab, and nine at the Lancaster University Phonetics Lab. Both labs have a similar recording setup and followed the same experimental protocol.

We acquired time-synchronized midsagittal ultrasound and audio data for all the speakers. The first 10 participants were recorded using the EchoB system from Articulate Instruments Ltd. Following a hardware update, the remaining participants were recorded using a Telemed Micro ultrasound speech system (Articulate Instruments Ltd), which can achieve a higher frame rate. A low-frequency probe was used for all recordings. The ultrasound settings varied from speaker to speaker. As a guiding principle for the settings, depth and field of vision were optimized to capture the available visual information, with the minimum possible cost to the frame rate. The resulting sampling rate of the ultrasound system ranged between 59.5 and 101 frames per second. The median sampling rate was 81.3 frames per second.

The participants wore a light-weight UltraFit headset (Spreafico et al., 2018) to stabilize the probe during the recording. After the headset was fitted, the participant was asked to produce a hyper-articulated /a/ so as to push the probe downward, and therefore avoid further probe movement during the recording. Next, we imaged the participant’s hard palate by asking them to swallow water (Epstein & Stone, 2005), and we imaged the occlusal plane, by asking them to bite on a disposable tongue depressor (a modification of the work by Scobbie et al., 2011). The participants then read the prompts from a screen in a pseudo-randomized order. Typically, between four and six repetitions of the experimental material were recorded per participant. The number of repetitions were guided by the feedback from the participant regarding their fatigue and by the time elapsed such that no recording would last longer than 40 min. Despite the use of head stabilization, there were some instances of headset movement. We noted these and discarded parts of the experimental material post hoc, as appropriate (see Section 2.4).

The time synchronization between ultrasound and audio was controlled by the Articulate Assistant Advanced software version 2.17. The audio data were captured using a Røde Micon lavalier microphone (Manchester) and sampled at 22,050 Hz (Manchester), or a Beyerdynamic Opus 55 microphone and sampled at 44 kHz (Lancaster). In addition to the ultrasound and audio data, co-registered articulography data were additionally collected from six participants, as described in the article by Kirkham et al. (2023). Only the ultrasound data were used in the current study.

The study received an ethical approval from the University of Lancaster Ethics Committee (ref. no. FL18188) and from the University of Manchester’s Proportionate University Research Ethics Committee (ref. no. 2022-13946-22714).

### 2.4 Data processing

Overall, 1,927 tokens of lax vowels were recorded. Seventy tokens (3.6% of the data) were subsequently discarded because they were affected by probe movement clearly visible in the resulting data. This left 1,857 tokens for analysis.

The audio data were force-aligned with Montreal Forced Aligner (McAuliffe et al., 2017), using the English language model and the American English dictionary. The boundaries were subsequently checked and manually corrected, as required. The onset of the vowel was placed at the offset of burst of the initial /b/. The vowel offset was marked at the beginning of closure for the final /d/.

The ultrasound data were processed using DLC implemented within Articulate Assistant Advanced, version 2.20, as described in Section 1.3. DLC is trained using manually selected labels that correspond to consistent anatomical landmarks in the midsagittal tongue view. These labels are chosen to correspond to specific features observed in the image and are not limited to surface edge features. DLC identifies the tongue surface landmarks as part of a surface contour represented by a 2D spline, utilizing all available data in the image with reference to the training set. This surface contour can be exported as a series of Cartesian or polar points relative to a designated origin.

The Cartesian coordinates of the DLC-identified landmarks were rotated on the occlusal plane (see Figure 1 for an illustration of the rotated occlusal plane) and exported for statistical analysis. The Cartesian coordinates were scaled by the software to represent real distances, measured in mm. These data were then reduced to a single time point per token, corresponding to the acoustic midpoint of the vowel. The choice of the acoustic midpoint was dictated by convenience, and it should be not be viewed as a principled approach to temporal reduction of articulatory vowel data. A principled approach, in our view, would be to identify the vowel target based on the kinematic properties of the relevant articulators. However, doing so would require identifying the relevant articulatory parameters that would serve as the basis for the identification of a target and performing a form of spatial reduction. Since our overall aim is to understand the implications of different types of spatial reduction, we did not want to make assumptions in this respect. Using the acoustic midpoint offers an imperfect but reasonable alternative in delivering a snapshot of articulatory relationships at a consistent time point.

Formant measurements were extracted dynamically using FastTrack (Barreda, 2021) running on Praat version 6.2.14 (Boersma & Weenink, 2009). They were subsequently reduced to temporal midpoint values, in line with the articulatory analysis.

### 2.5 Analysis

#### 2.5.1 Measures

The measures evaluated in this study are summarized in Table 1. We compared various measures of tongue position, operationalized as X-Y coordinates of anatomically defined points on the tongue, as well as the X coordinate of the highest point on the tongue, with two different normalization approaches to defining height. In terms of anatomical measures, previous studies have used the position of the tongue root or the tongue dorsum as correlates of tongue position. In this context, we considered the X-coordinate of DLC knots 2, 3, 4, 5, 6, and 7 as potential correlates (see Figure 1 for an illustration of the location of knots).

**Table 1.** Summary of the Tongue Measures Evaluated in the Study.

	Abbreviation	Definition
Tongue position measures	K2 X	X-coordinate of DLC Knot 2
	K3 X	X-coordinate of DLC Knot 3
	K4 X	X-coordinate of DLC Knot 4
	K5 X	X-coordinate of DLC Knot 5
	K6 X	X-coordinate of DLC Knot 6
	K7 X	X-coordinate of DLC Knot 7
	HP X	X-coordinate of highest point on the tongue
	HV X	X-coordinate of the highest vertex
Tongue height measures	K5 Y	Y-coordinate of DLC Knot 5
	K6 Y	Y-coordinate of DLC Knot 6
	K7 Y	Y-coordinate of DLC Knot 7
	HP Y	Y-coordinate of highest point on the tongue
	HV Y	Y-coordinate of the highest vertex
Tongue shape measures	TCP	Tongue curvature, as in Ménard et al. (2012)
	TCP	Tongue curvature position, as in Ménard et al. (2012)
	MCI	Modified Curvature Index, as in Dawson et al. (2016)

**Figure 3.**
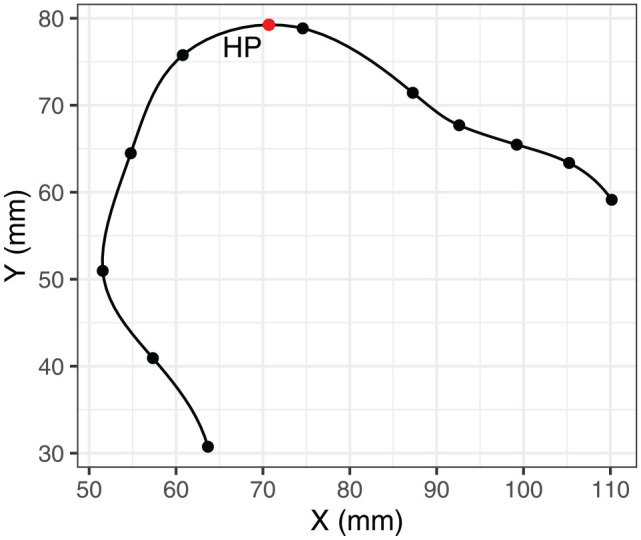
Example illustration of spline smoothing. *Note.* The black points on the spline contour represent original DLC knots. The estimated highest point (HP) is shown in red.

The highest point on the tongue was defined as the point on the tongue surface with the relatively highest value of the Y-coordinate. Recall that the orientation of the ultrasound data was normalized to the occlusal plane, so that X and Y coordinates are meaningfully related to high-low and front-back dimensions. The highest point need not necessarily overlap with any particular DLC point. To estimate the coordinates of the highest point, we smoothed the tongue splines and interpolated them to span 100 points (from original 11). Smoothing was done, using piecewise cubic polynomials, as implemented in the smoothr package (Strimas-Mackey, 2023). The coordinates are interpolated independently, which means that the curve always passes through the original DLC knots. Figure 3 shows an example of the smoothing relative to the original DLC knots, as well as the location of the highest point.

**Figure 4.**
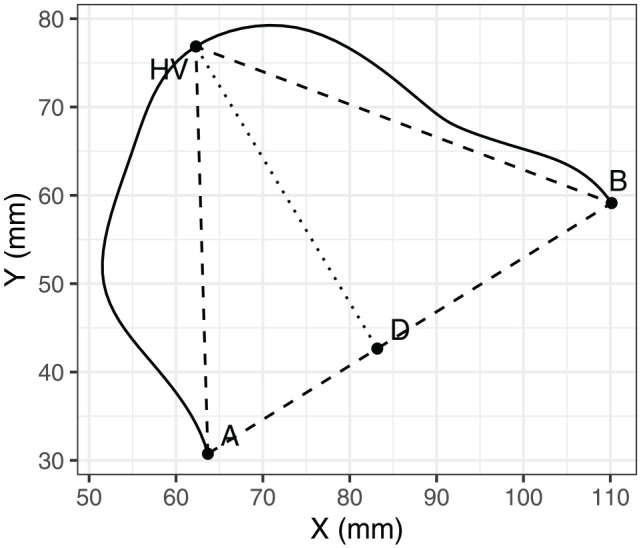
Illustration of tongue curvature measures based on the study by Ménard et al. (2012). *Note.* Tongue curvature position (TCP) is defined as HVD/AB, and tongue curvature (TC) is defined as AD/DB.

In addition, we included the X-coordinate of the highest point as defined by Ménard et al. (2012), that is, the point on the tongue surface that is farthest from the line joining the vallecula with the tongue tip. This is illustrated in Figure 4: vallecula corresponds to Point A, and tongue tip corresponds to Point B. We refer to the highest point according to this definition as the “highest vertex,” abbreviated as HV. We used smoothed spline data to estimate the HV. We also use the smoothed splines for the illustration of example tongue contours through the rest of the paper.

As far as measures of tongue height are concerned, previous studies using anatomically-defined feature typically choose the tongue dorsum to capture tongue height. We therefore considered the Y coordinate of DLC knots 5, 6, and 7 as potential correlates of tongue height. In addition, we used the Y-coordinate of the highest point on the tongue and Y-coordinate of the HV as potential height measures.

Finally, we considered three potential measures of tongue shape, as an alternative to quantifying tongue height. The first two measures are TC and tongue curvature position (TCP), as proposed by Ménard et al. (2012). These measures are based on a triangle fitted to the tongue contour, where the base of the triangle joins the edges of the tongue contour, with the opposite edge of the triangle representing the HV, as shown in Figure 4. In this triangle, A corresponds to the vallecula, B corresponds to tongue tip, and HV represents (the point on the tongue surface farthest removed from the base of the triangle). TCP is defined as HVD/AB, and TC is defined as AD/DB. The third shape measured we considered was the Modified Curvature Index (MCI), as defined by Dawson et al. (2016). Specifically, MCI represents the integrated curvature values with respect to the arc length of the tongue.

A series of statistical analyses were then carried out to compare the different measures, as described below in Sections 2.5.2–2.5.4. The statistical analysis was carried out in R, version 4.4.1 (R Development Core Team, 2024).

#### 2.5.2 Multidimensional scaling and Procrustes analysis

The aim of this part of analysis was to address Research Question 1: How well do different measures capture the overall midsagittal lingual contrast between vowels? More specifically, this analysis aims to establish whether a combination of two physical measures, representing tongue position and tongue height/shape, can successfully replicate the overall lingual distance between vowels. To capture the overall distance between vowels, we submitted the X-Y coordinates of the 11 DLC points to multidimensional scaling (MDS). MDS is a technique for dimensionality reduction, similar to principal component analysis. However, an important difference between the two approaches is that MDS focuses on preserving the pairwise distances between data points, and thus, it works well to capture the relative articulatory distance between vowels. Using MDS, we reduced the input coordinates to two dimensions that best represent the pairwise distance between vowels. We then compared the shape of the vowel spaces defined by MDS1 and MDS2 to two-dimensional vowel spaces constructed using physical measures of tongue position and height/shape. Through this comparison, we tried to determine which physical vowel spaces are the most representative of overall lingual vowel contrast.

**Figure 5.**
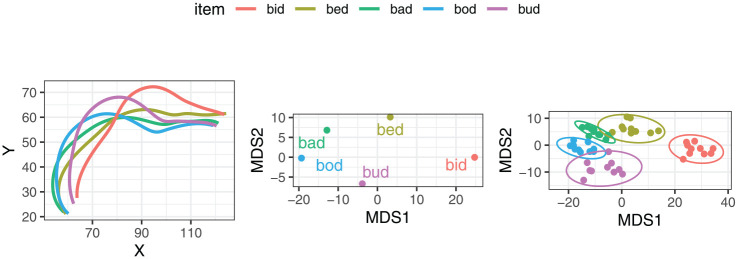
Illustration of the input and output of multidimensional scaling for an example speaker, LNCS2. *Note.* The left panel shows a single set of tongue spline data interpolated across the 11 DLC knots. The mid panel shows the corresponding MDS1 and MDS2 values for these tokens. The rightmost panel shows the MDS values for the same speaker across all repetitions.

The input and output of MDS for an example speaker are illustrated in Figure 5. The leftmost panel of the figure shows the midsagittal tongue contours for a single token of the five vowels, as produced by speaker LNCS2. The central panel shows the corresponding values of the two MDS dimensions for the same vowel tokens. The rightmost panel shows the MDS dimension values for all the repetitions produced by the same speaker.

Impressionistically, MDS reduction provides a close correspondence to the relative position and height of the tongue as visible in the tongue splines, although a rotation is involved, as well as a reasonably close match to acoustic/auditory qualities of those vowels. The MDS dimensions are based on a PCA (principal component analysis) rotation, such that MDS1 corresponds to a vector with the largest variance in the data. In this case, MDS1 represents the articulatory diagonal between *bid* and *bod*, the two most distinct vowels in the set. MDS2 represents the vector with the second largest variance in the data. For this particular speaker, the most extreme items on the MDS2 scale are *bud* and *bed*. The rightmost panel indicates the variance within the individual categories. Some degree of variance is present, suggesting a certain amount of error, the possible sources of which include headset movement, error in DLC tracking, and natural variation in production.

**Figure 6.**
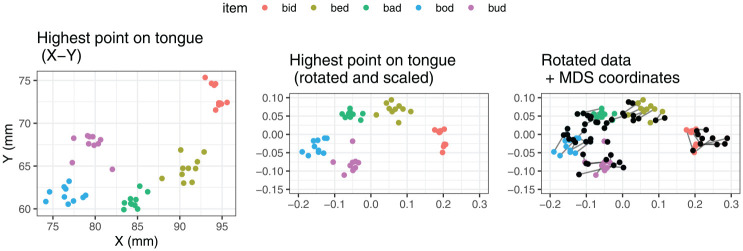
Illustration of the Procrustes analysis for an example set of measures, X-Y coordinates of the highest point on the tongue for speaker LNCS2.

The dimensionality reduction was performed separately for each speaker in the dataset. The MDS-based vowel spaces were then used as a set of baselines against which we compared 2D vowel spaces created by pairwise combinations of physical measures of tongue position and height. To do this, we first carried out Procrustes analysis, which rotates, scales, and translates the physical 2D vowel spaces so that they match the reference MDS space as closely as possible. The input and output of the Procrustes analysis are illustrated for speaker LNCS2 in Figure 6, using the X-Y coordinates of the highest point on tongue as an example. We then calculated the residual error, quantified as the sum of squares between the rotated physical dimensions and the reference MDS values. The lower the sum of squares, the better the match between the physical measures and the MDS dimensions in terms of capturing the distances between the vowels. In this context, we carried out a comparison of the measures to establish which ones are associated with the lowest sum of squares.

The Procrustes analysis was carried out for each pairwise combination of possible position and height measures (64 combinations altogether), separately for each speaker. MDS and Procrustes analysis were performed using the vegan package in R (Oksanen et al., 2022). The results of this analysis are reported in Section 3.1.

#### 2.5.3 Linear discriminant analysis

The next set of measure comparisons was carried out to address our second research question: ‘How consistent are the measures across speakers’? The analysis was based on a classification task which evaluated whether models trained on different pairs of combinations of tongue shape and height / position measures could perform correct phonemic classification of tongue shapes in data from unseen speakers. The use of a classification task is similar to Dawson et al. (2016).

We used Linear Discriminant Analysis for the classification. First, we identified possible pairs of articulatory measures, each time combining a possible measure of tongue position with a measure of tongue height / shape. There were 64 possible combinations. For each of those combinations of measures, the algorithm was trained to classify the five vowels, based on data from 30 speakers (75% of the sample). We then tested the accuracy of the classification using the data from 10 unseen speakers. For each combination of measures, the procedure was repeated 100 times with resampling. The measures were scaled within speakers for this task, to reduce the variability associated with anatomical differences and with differences in the center of coordinate origin.

We compared the overall accuracy values across the different measures, with accuracy defined as the number of correctly classified phonemes divided by the number of trials. We further performed an analysis of errors, using a generalized linear mixed model. The model was predicting the likelihood of an error, depending on an interaction between phoneme and the measure of tongue position, and phoneme and the measure of height / shape, with random intercepts for client and trial. The results of the analysis introduced in this section are presented in Section 3.2.

#### 2.5.4 Relationships between the individual measures

Finally, we carried out an exploratory analysis that aimed to address our third research question: “What information about vowel contrasts is replicated across different measures, and what information is unique to specific measures?” The intention here is to shed more light on the measures, the relationship between the different measures, and the type of information they convey about vowel contrasts. As we took multiple measurements from the tongue surface, we can expect that all the measures will be correlated with one another to some degree. However, it is important to establish the extent of such correlations, to understand the full methodological implications of using different measures.

In this part of the analysis, we mainly relied on correlation analysis. All the values were scaled within the speaker prior to extracting the correlation coefficient and reduced to a by-speaker by-item mean, to reduce the autocorrelation within the data. Pearson’s correlation coefficient was then calculated for all the relevant pairs.

In order to illuminate the relationship between different articulatory measures and the acoustic output, we calculated the correlations between the individual measures and the first two vowel formants, scaled within speaker. We do not expect perfect correlations because different speakers may manipulate their tongue differently to achieve the target resonances, depending on their individual vocal tract shape. Nevertheless, it is instructive to know how strong the relationships are, in the context of the wider debate on the role of the tongue in vowel production, as discussed in Section 1.1. Broadly, we expect the first formant to be inversely correlated with tongue height, and the second formant to be correlated with tongue position, such that more front vowels have a higher F2. Some non-linearities may arise; for example, there may be a sharp rise in F1 in low vowels (compared to high vowels) due to the increase in the volume of the back cavity. In general, non-linearities may be present when there is a very wide or very narrow constriction, or more than one constriction. The results of the correlation analysis are reported in Section 3.3.

### 2.6 Data access statement

The data (measurements) and code used for analysis reported in this study are available as an Open Science Framework repository at https://osf.io/hdpmb/.

## 3 Results

### 3.1 Evaluation of individual measures for capturing vowel distances

As described in Section 2.5.2, we conducted by-speaker MDS, using the X-Y coordinates of all DLC knots, to obtain reference two-dimensional vowel spaces. We first analyze the stress values produced by the MDS. This is done to establish whether the two MDS dimensions provide a good representation for the overall lingual contrast between vowels. The median stress value was 0.04, and the upper quartile was 0.057, which means that the fit was typically good, according to the threshold proposed by Kruskal (1964). We can therefore generalize that two degrees of freedom are sufficient to capture the overall tongue movement relevant to vowel contrasts, in line with previous research that used similar dimensional reduction approaches (see Section 1.2).

To establish whether the information captured by the abstract MDS dimensions can be obtained by using a combination of physical measures, we applied the Procrustes analysis to project the physical coordinates onto the MDS vowel space, and we analyzed the sum of squares, that is, the residual distance between the rotated physical coordinates and the MDS values. The smaller the sum of squares is, the better the physical measures are at capturing the overall lingual contrast.

Table 2 lists the 10 out of 64 possible combinations of measures that had the lowest mean sum of squares (the mean is a by-combination mean across all the speakers). The best combination overall was the combination of X and Y coordinates of Knot 7 (anterior part of the dorsum). However, as we can see in Table 2, multiple combinations returned an only incrementally higher sum of squares. Thus, this analysis does not deliver a clearly preferred combination of measures, but rather, a set of possible combinations.

**Table 2.** The Ten Combinations of Position and Height/Shape Measures With the Lowest Mean Sum of Squares.

Position measure	Height/shape measure	Sum of squares
K7 X	K7 Y	0.15
K6 X	K7 Y	0.161
K5 X	K7 Y	0.166
K7 X	HP Y	0.169
K4 X	K6 Y	0.172
K4 X	K7 Y	0.173
K4 X	K5 Y	0.179
K5 X	K6 Y	0.181
K7 X	K6 Y	0.181
K6 X	HP Y	0.183

*Note.* HP: highest point.

**Figure 7.**
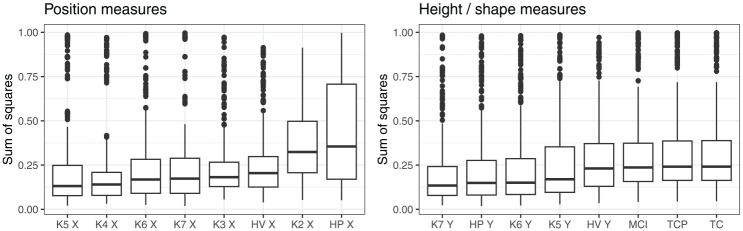
Sum of squares (residual error), depending on position and height/shape measures. *Note.* Lower values indicate better match between particular measure and an abstract overall measure of lingual contrast.

Figure 7 shows the distribution of sum of squares depending on the measure. In this case, we compare values per measure across the possible combinations with multiple other measures. In each panel, the measures are arranged in the ascending order according to the median sum of squares. The comparison suggests that some position measures clearly fare worse than others. In particular, the position of the highest point on the tongue and the position of Knot 2 (tongue root) are relatively worst at capturing the overall position contrast between vowels. However, no measure of position emerges as a clearly preferred one. The four measures with the smallest median values, that is, X coordinates of K5, K4, K6, and K7, produce very similar results. This would suggest that measuring the tongue position anywhere at the tongue dorsum, or at a point somewhat posterior to the tongue dorsum, generates similar levels of residual error when it comes to capturing the tongue position contrast.

When it comes to measures of height/shape, we can generalize that shape measures are associated with slightly higher residual error than height measures, except the height of the HV, which performs similarly to the shape measure. However, no clear preference is evident when it comes to the other possible measures of tongue height. As seen in the right panel of Figure 7, the level and distribution of residual error are similar, when comparing the height of DLC knots 7, 6, and 5 and the height of maximum tongue height (HP Y).

In summary, this analysis suggests that there are several measures of tongue position and height that can capture the overall lingual contrast with a similar degree of accuracy. For tongue position, the position of the tongue dorsum is broadly a better measure, compared to the position of tongue root, the position of the highest point on the tongue, or the position of the HV. Several measurement points on the tongue dorsum generate similar residual error as each other, and thus seem equivalent. Analogously, several tongue height measures we considered perform similarly. However, whether or not these measures are genuinely equivalent depends not just on the magnitude of error they generate but also on the nature of error they produce. In this context, we compare the measures further in Sections 3.2 and 3.3.

### 3.2 Variability of measures across speakers

This section corresponds to the analysis described in Section 2.5.3. Having considered how well pairwise combinations of physical measures capture the overall lingual contrast, we subsequently analyzed the invariance of measures across speakers. This was evaluated using a classification task, in which linear classifiers trained on pairs of physical measures were tested by performing phonemic classification using unseen articulatory data (see Section 2.5.3). Relatively higher accuracy values would indicate that the measure is relatively stable across speakers.

The overall mean classification accuracy was 61%, which suggests a considerable level of error. In comparison, the same task performed using normalized formant values delivers a mean accuracy of 95%. This discrepancy might be indicative that articulation is overall more variable than acoustics, or that different articulators, such as the lips, contribute to the acoustic differentiation of vowels. While this generalization is largely in line with our impression of the data, comparing variability between articulation and acoustics is not entirely straightforward because the measurement error is potentially different between the two domains. Table 3 shows the mean accuracy of classification (across multiple pseudo-randomized speaker samples) for different pairwise combinations of measures of tongue height and tongue shape/position. The table lists the 10 combinations with the highest mean accuracy. The best combination in this case was the X-coordinate of Knot 4 and Y-coordinate of Knot 6. However, similar to the sum of squares comparison, the accuracy levels were very similar across different combinations of measures. The top 10 ranking combinations vary between 70% and 67% in accuracy.

**Table 3.** The Ten Combinations of Position and Height/Shape Measures With the Highest Classification Accuracy.

X1 X2 accuracy		
Position measure	Height/shape measure	Accuracy
K4 X	K6 Y	0.7
K3 X	TC	0.698
K5 X	K7 Y	0.696
K5 X	K6 Y	0.687
K4 X	HP Y	0.685
K3 X	K5 Y	0.684
K4 X	K5 Y	0.683
K4 X	HV Y	0.677
HV X	HV Y	0.674
K4 X	K7 Y	0.673

*Note.* HP: highest point; HV: highest vertex; TC: tongue curvature.

**Figure 8.**
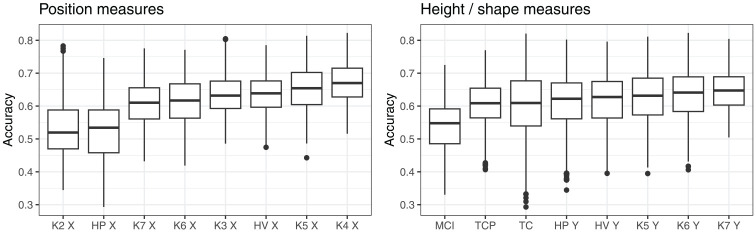
Overall accuracy of classification, depending on position and height measures.

Figure 8 shows how the overall classification accuracy varies, depending on the measure of position and height/shape. The accuracy was overall lowest when the X-coordinate of K2 (tongue root) was used, closely followed by the X-coordinate of the highest point on the tongue. The accuracy was highest when the position of knots 4 or 5 was used (the part of tongue directly behind the dorsum and the posterior part of the dorsum). The accuracy dropped off, but only slightly, when the position of the HV, or position of the tongue root (K3), was used.

As far as the measures of height/shape are concerned, the accuracy was generally lower for the shape measures, MCI, TCP, and TC, compared to the measures of height (although Table 3 suggests that some shape measures did relatively well when combined with position of the tongue root). Among the height measures, the height of knots 7, 6, and 5 produced similar classification accuracy, and there was only a small drop in accuracy for the maximum tongue height.

We further considered how the likelihood of error was affected by the interaction between the measure and the item. We fitted a generalized linear mixed model predicting the likelihood of error (incorrect classification) depending on an interaction between the target vowel phoneme and the position measure, and an interaction between the target vowel phoneme and the height/shape measure. The model also included random intercepts for speaker and trial.

**Figure 9.**
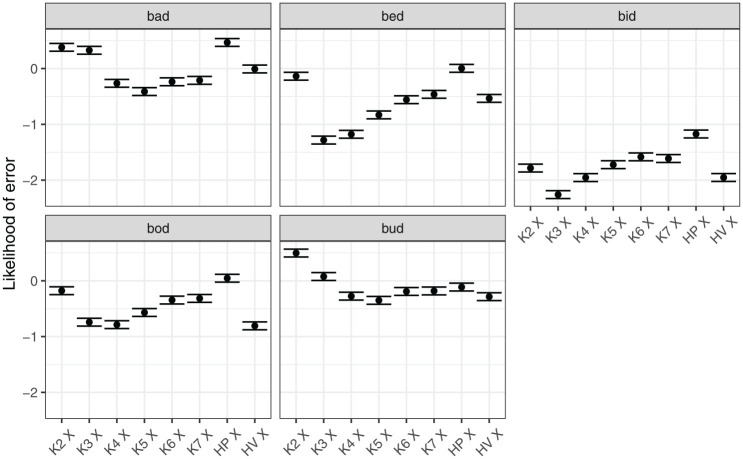
Model predictions for likelihood of error depending on the position measure within a vowel.

Figure 9 shows model predictions for the interaction between the measure of tongue position and vowel. The likelihood of error varied substantially between vowels, with *bid* overall least likely to be misclassified, and *bad* being the most likely one. Furthermore, different measures of position affected the likelihood of error differently for different vowels. Knots 3 and 4 were associated with the smallest likelihood of error for front vowels, *bid* and *bed* (although measure differences within *bid* were small). In contrast, the more posterior Knot 5 was the best measure for the classification of *bad* and *bud*.

**Figure 10.**
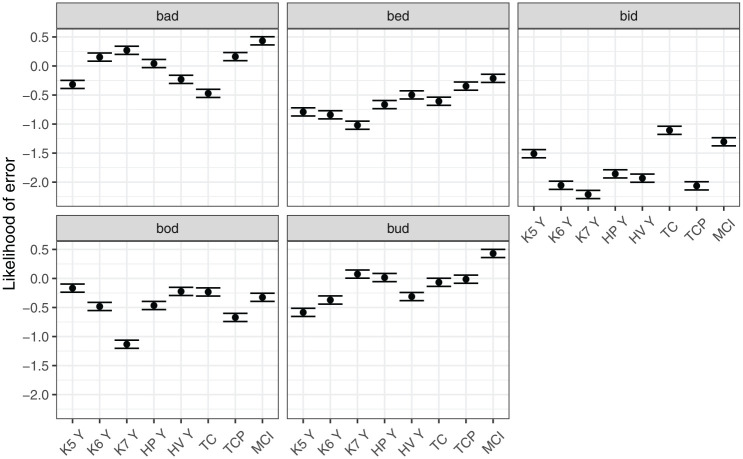
Model predictions for likelihood of error depending on the height measure within a vowel.

Figure 10 shows model predictions for the interaction between measure of tongue height/shape and vowel. We can generalize that the likelihood of classification error was lower when Knot 7 (anterior part of dorsum) was used to classify vowels *bid, bed*, and *bod*. However, the same knot was associated with relatively high level of error for *bud* and *bad*. For these vowels, the best height classification was height of Knot 5, that is, the posterior part of the tongue dorsum. In addition, for *bad*, tongue curvature was a measure associated with relatively low likelihood of error.

In summary, the classification task delivers globally similar results to the analysis of Procrustes sum of squares. No particular pairwise combination of measures was found to be clearly superior. However, the task can differentiate between the average performance of measures to some extent, returning several preferred measures of tongue position and tongue height/shape. In addition, the error analysis reveals that performance of measures varies depending on the vowel, suggesting that the stability of vowel contrasts is different for different parts of the tongue, depending on the vowel.

### 3.3 Correlation between measures

The analyses reported so far suggest that multiple measures of tongue position perform similarly in capturing lingual contrast, and they also show relative stability across speakers. This is especially true of the position (X-coordinate) of knots 4 and 5, closely followed by the position of knots 6 and 7. For tongue height, four measures fared relatively well across the two analysis: the height of knots 5, 6, and 7 and the maximum tongue height. As the next step, we considered to what extent these measures are correlated with one another, and to what extent they offer complementary information. The results reported in the following paragraphs correspond to the analysis introduced in Section 2.5.4.

**Figure 11.**
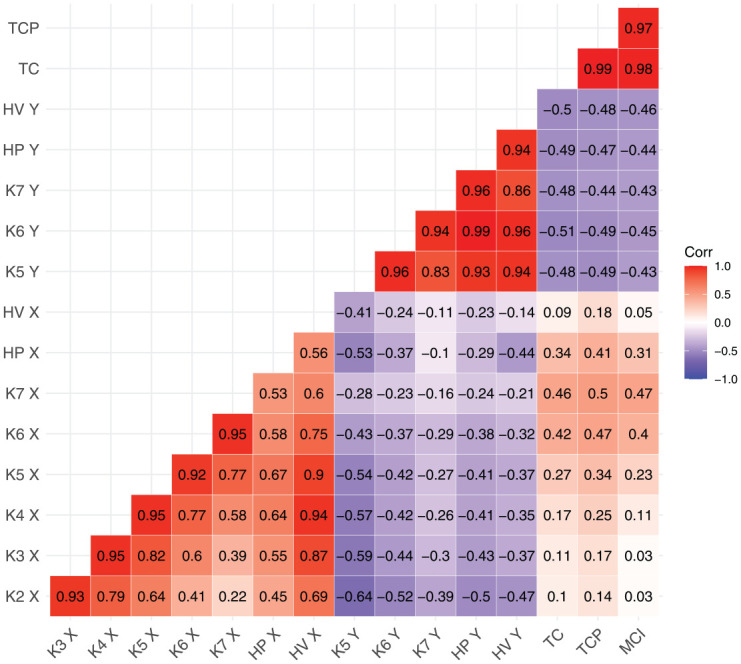
Correlation matrix showing correlation coefficients for all possible pairwise combinations of measures in this study.

Figure 11 summarizes the correlation coefficients between all possible pairs of measures (the measures were scaled, and by-speaker by-item means were used as the input to the correlation tests). Overall, the various tongue position measures are correlated with one another. A cluster of correlations also emerges for tongue height measures, and another one for tongue shape measures. As expected, the correlations are strongest between neighboring knots, for example, the correlation coefficient for the position of Knot 4 and Knot 5 is .95. However, the strength of the correlations drops off considerably for pairs of non-adjacent knots. For example, the correlation between the position of Knot 4 and Knot 7 is .58. This would suggest that those two position measures capture different pieces of information.

Similarly, we find varying correlation strengths between the four preferred measures of tongue height. Maximum tongue height shows very strong pairwise correlations with the height of knots 5, 6, and 7 (all above .9). However, the correlation between the height of Knot 5 and the height of Knot 7 is somewhat weaker at .83. We also note that the shape measures show medium levels of correlation with the measures of tongue height, as well as with some measures of tongue position, especially K6 X and K7 X. This suggests that tongue shape combines some information about tongue position and height.

**Figure 12.**
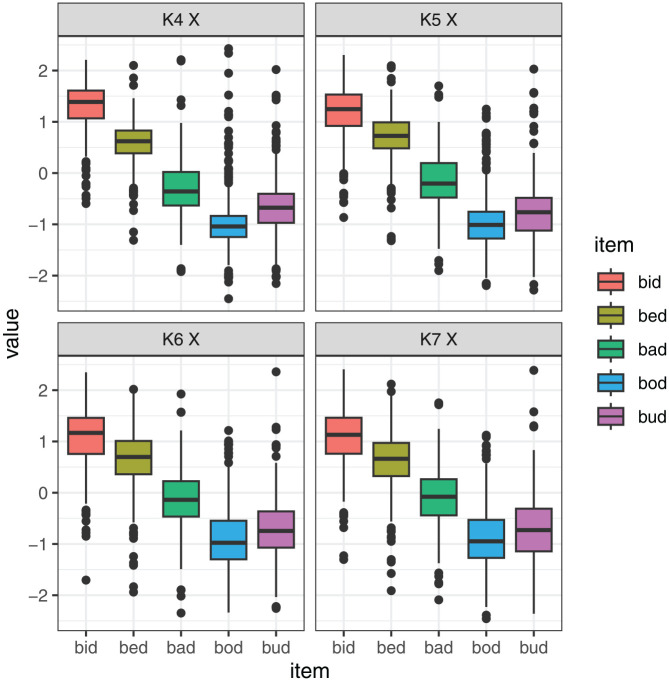
Scaled values of X-coordinate by vowel for four selected DLC knots (four different measures of tongue position).

**Figure 13.**
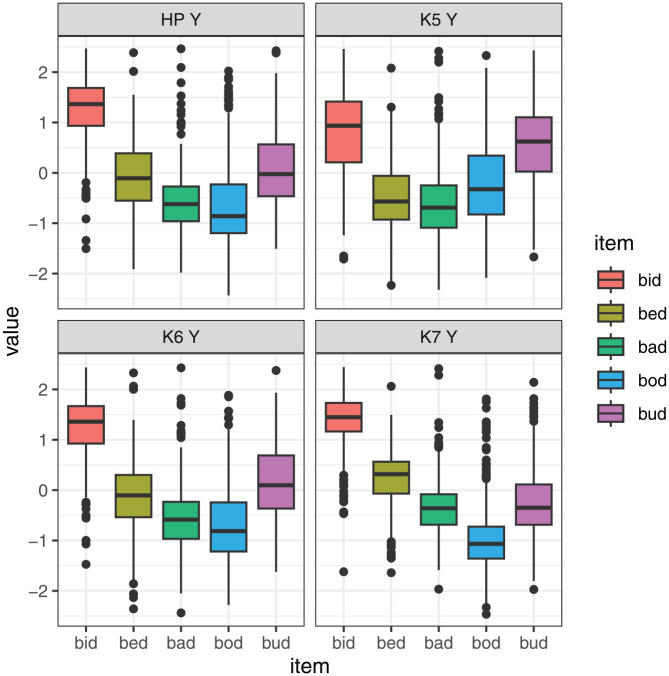
Scaled values of X-coordinate by vowel for three selected DLC knots at the maximum tongue height (four different measures of tongue height).

All in all, we find that the positions of different points on the tongue are largely correlated with one another, as expected, but the correlations are not perfect, and the same is true for tongue height. Figures 12 and 13 further explore the nature of these differences. Here, we focus on selected measures that performed relatively best in the Procrustes analysis and discrimination analysis. The two figures show the value of the selected measures (scaled within speaker), depending on the item. The illustrations of the individual measures are generated using the same data, but they provide a somewhat different view of the data. The differences are fairly subtle for the tongue position measures, plotted in Figure 12, but we can nonetheless observe that particular measures are more sensitive to particular contrasts. For example, *bud* and *bod* are clearly contrastive at Knot 4, but their position largely overlaps at Knot 7. Conversely, the *bad* vs. *bud* position contrast is larger at Knot 7 than at Knot 4.

Differences between measures are even more noticeable, comparing the relative value of four measures of tongue height, as shown in Figure 13. For example, the height contrast between *bid* and *bud* is very large at Knot 7 and fairly small at Knot 5, and it has an intermediate value if we look at the maximum tongue height. Meanwhile, *bad* and *bud* have similar height at Knot 7, but they are contrastive at Knot 5. Clearly, measuring tongue position and height at different points on the tongue provides a very different view of the levels and magnitude of vowel contrasts.

Finally, we also analyzed the correlation between different measures and vowel formants (F1 and F2). Similar to the correlations between measures, we used means of values scaled within speaker, both for articulatory measures and formant measures. The correlation coefficients are summarized in Table 4. Generally, very strong positive correlations could be observed between F2 and multiple measures of tongue position. The highest correlation coefficient (*r* = .941) was observed for the X-position of Knot 5 and F2, but the X positions of knots 4, 6, and 7 also showed very strong correlations with the second formant, with *r* greater than .9, as did the X position of the HV. Among position measures, the relatively weakest correlation was found for the X position of K2, but it was nonetheless strong with *r* = .7. The hierarchy of how well tongue position measures correlate with F2 is very close to the results of the Procrustes analysis and classification analysis. All in all, the second formant is very well predicted by the relative position of multiple points on the tongue, especially the tongue dorsum.

**Table 4.** Correlation Coefficients Between Scaled F1 and Scaled Articulatory Measures (Left Column) and Between Scaled F2 and Scaled Articulatory Measures (Right Column).

Measure	Correlation With F1	Measure	Correlation With F2
K5 Y	−.714	K5 X	.941
HV Y	−.659	K4 X	.93
K6 Y	−.622	K6 X	.921
HP Y	−.602	HV X	.914
K2 X	−.549	K7 X	.91
TCP	−.497	K3 X	.874
K7 Y	−.396	K7 Y	.831
K3 X	−.381	TC	−.796
MCI	.369	HP X	.77
TC	−.243	TCP	.719
HV X	−.218	K2 X	.698
K4 X	−.202	HP Y	.568
HP X	.1	K6 Y	.56
K5 X	−.081	HV Y	.456
K6 X	−.066	MCI	−.433
K7 X	−.062	K5 Y	.119

*Note.* HP: highest point; HV: highest vertex; TC: tongue curvature; TCP: tongue curvature position; MCI: Modified Curvature Index.

In comparison, the correlations between scaled measures of tongue height and scaled F1 were weaker across the board, and they were markedly different from other comparisons. This suggests that, unlike F2, F1 does not have a single robust midsagittal lingual correlate in this set of vowels. The relatively strongest negative correlation was found for the height of Knot 5; however, with *r* = −.714, this was at the level of weakest correlation for position–F2 relationship. According to the magnitude of the correlation coefficient, the second-ranking measure for (negative) correlation with F1 was the Y-coordinate of the HV. The third in line was height of Knot 6, also closely correlated with the height of Knot 5.

### 3.4 Summary of the results

We performed different types of evaluations to compare various measures of tongue position and measures of tongue height/shape. The comparison was guided by three research questions, concerned with identifying the most optimal measures for capturing midsagittal lingual vowel contrast, the measures that are most consistent across speakers, and illuminating the relationship between different measures. The evaluations we used included comparing physical measurements to abstract distances between vowels, representing different levels of contrast, unseen vowel phoneme classification, and correlational and descriptive analysis. Synthesizing findings from different strands of analysis aimed at addressing the individual questions, we observe somewhat consistent results for measures of tongue position, but less so for measures of tongue height/shape.

With respect to position measures, the position of knots 4 and 5 (the posterior part of the tongue dorsum toward the tongue root) generally emerges as the most representative of position contrasts, and most consistent across different speakers. The X-coordinates of knots 4 and 5 are strongly correlated with each other, as well as with F2, and thus, these two measures of tongue position are equivalent. In contrast, comparing the measures of tongue height and shape delivers less consistent results. Four measures fare somewhat better than others: the heights of knots 7, 6, and 5 and the maximum tongue height. However, these measures cannot be said to be equivalent, as they afford a strikingly different view of height contrasts. We provide possible explanations for these findings in Section 4, and we consider their implications for articulatory methodology and interpretation of articulatory findings.

## 4 Discussion

In this study, we investigated whether two-dimensional models of vowel articulation can be used to substantiate physical measures of the tongue derived from articulatory imaging methods. We focused specifically on the tongue-arching model, where the primary dimensions are tongue position and height, and a muscle-based model, where the dimensions are tongue position and tongue shape. Our results suggest that two dimensions are, in principle, sufficient to capture the lingual contrast between vowels, or at least that is the case for a simple and well-dispersed five-vowel subsystem that we considered. The generalization that two degrees are enough follows from the observation that two abstract dimensions obtained via MDS capture most of the variance in the underlying data. However, our results also suggest that two dimensions of lingual vowel contrast do not have robust and reliable physical correlates across different vowels.

We considered a range of potential measures representing tongue position, height, and shape. We evaluated how well these measures capture the overall lingual distance between vowels as derived bottom-up using MDS. Second, we investigated whether these measures can serve as the basis of phoneme classification in unseen articulatory data, thereby indicating a level of invariance across speakers. The two approaches broadly converge in providing a hierarchy of measures. Among the measures of position, the X-coordinate of the tongue dorsum measured at Knot 4 and 5 emerges as preferred, closely followed by measurements of X-coordinate at knots 6, 7, and 3, although some differences are apparent here between the two evaluation approaches. At the other end of the scale, the X-coordinate of the highest point on the tongue, the X-coordinate of the HV, and the X-coordinate of Knot 2 (tongue root) are clearly dispreferred. Our approach also allows us to establish some hierarchy across the potential measures of tongue height and tongue position. Generally speaking, the shape measures we used performed worse compared to height measures. Among the measures of height, the preferred ones seem to be the heights of knots 7 and 6, as well as the maximum tongue height, with the height of Knot 5 as a further potential high-ranking measure.

These findings could potentially be taken to indicate that there are multiple measures of tongue position and height that work equally well, and that are therefore equivalent. However, while multiple possible combinations of measures deliver similar levels of residual error, they are not in fact equivalent because they offer a different view of position and height differences. This is especially true of the height dimension. We find similar levels of error associated with measuring tongue height at the central part of the tongue dorsum, the anterior part of the tongue dorsum, and at maximum tongue/dorsum height. However, each of those measurements involves a different bias that systematically skews the residual error. In general, height measurements taken at the anterior part of the dorsum underestimate the height of back vowels, whereas measurements taken at the posterior part of the dorsum underestimate the height of front vowels. Measuring maximum tongue height might seem like a good compromise in this context. However, as we have seen, maximum tongue height is not uniquely representative of the overall lingual contrast, and it is fairly variable among speakers. Therefore, there is no strong basis for singling it out as the preferred correlate of tongue height. One might hypothesize that the maximum tongue height is perhaps especially relevant to vowel resonances. This, however, is not the case, as the best correlate of F1 is in fact the height of the posterior part of the dorsum.^1^

Our overall interpretation is that the articulatory metaphor of retraction and raising is broadly correct in capturing the relationship between different vowels, but the optimal measures of articulatory retraction and raising may be different for different vowels. This is consistent with the muscle-based model of vowel production by Honda (1996), in which different muscles are involved in the production of different vowels, resulting in the constriction being formed in a different part of the vocal tract. As a result, the retraction and fronting contrasts hold as expected for some part of the tongue, but they do not systematically represent any particular point on the tongue, be it defined in terms of an anatomical location or the location of the constriction.

**Figure 14.**
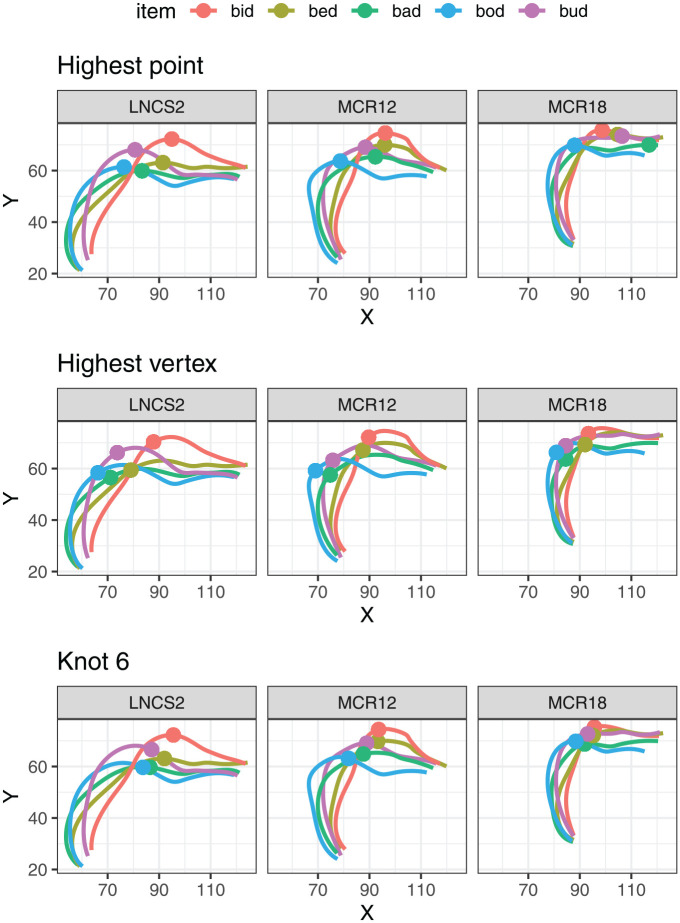
Tongue splines representing a single repetition of the five tokens for three example speakers, along with the location of three possible points measures: highest point on the tongue, highest vertex, and DLC Knot 6.

Having presented systematic evidence concerning issues with single-point reduction of whole tongue contours, let us also consider some specific examples that help illustrate the same point. Figure 14 shows the outline tongue contours for a single set of repetitions of the five vowels, produced by three example speakers. The same tongue contours are plotted three times, overlaid with a different point coordinates in each case. The top panel shows the location of the highest point on the tongue. This measure appears to work reasonably well for speaker LNCS2, but obvious issues transpire for the other two speakers, either as a result of a relatively flat shape or coarticulation with the following coda /d/. In some cases, the location of the highest point on the tongue is too anterior, clearly not representing the vocalic constriction. This might be mitigated by restricting the search area for the highest point to the tongue dorsum, as opposed to the whole tongue. However, for relatively flat tongue shapes (e.g., *bud* pronounced by MCR18), doing so would still result in some arbitrary variation in the location of the highest point. This type of issue is likely the reason why we generally found that the height of the highest point was a fairly good height measure, but the location of the highest point was not a reliable measure of tongue position.

An alternative approach is to define the highest point relative to the line joining the edges of the tongue contour (see Figure 4), rather than the occlusal plane. We have been referring to this point on the tongue as the HV. As shown in Figure 14, the location of the HV is more robust against coarticulation and variation in tongue shape, compared to the highest point. Even so, it is not clear that this measure consistently captures tongue height. For example, the location of HV seems quite arbitrary for *bed* across the three speakers. HV is also consistently quite posterior for high-front vowels (*bid*), possibly underestimating tongue height in these cases.

Focusing on a single DLC knot introduces a different set of problems. The bottom panel of Figure 4 illustrates the location of DLC 6, that is, the central part of the tongue dorsum for the five vowels in the three speakers. The height of Knot 6 seems to represent the height contrast quite well for these three speakers. However, the location of this point would appear to underrepresent the extent of position contrast, especially for LNCS2, for whom the constriction is clearly more posterior in *bud* and *bod*.

A final important point of the comparison delivered by Figure 14 is that the three sets of coordinates—highest point, HV, and Knot 6—deliver a very different representation of the vowel contrasts. Individually, these representations may appear sensible, and we could find examples of speakers for whom the representation matches the impression of inter-vocalic contrasts that we may form based on a visual inspection of the tongue splines. However, once we take multiple speakers and multiple measures into account, it becomes clear that no single two-dimensional model can offer an adequate methodological solution to the problem of quantifying tongue articulation. This conclusion closely matches the results of a quantitative comparison of measures undertaken in this study, and it furthermore suggests that findings delivered by studies that rely on different measures of tongue height/shape are not directly comparable and may require a careful interpretation that takes the properties of the specific measure into account.

Some of the issues we have discussed are likely to be compounded when vowel dynamics come into play. In particular, dynamic tracking of tongue height based on the change in Y coordinate of the highest point on the tongue is ill-advised because the location of the highest point shifts over time from the tongue dorsum to the tongue tip. Based on an exploratory analysis of selected examples (available in the OSF repository), such shifts can be quite abrupt. In comparison, dynamic tracking of DLC knots does represent displacement of the same point on the tongue, and it is conceptually similar to standard practice in articulometry. From the dynamic point of view, DLC knots present a more reliable measure than the highest point, but we must bear in mind that different knots are key to different vowels.

Given the complexity of interpretation, one may ask if there is much to be gained from articulatory studies of vowels, especially where sociophonetic comparisons are concerned. Most of the time, articulatory data are very noisy, and when they are not, the relevant patterns may turn out to be acoustically systematic and somewhat predictable (Noiray et al., 2014; Whalen et al., 2018). Without doubt, acquiring articulatory data can be a lot of work for little gain where some research questions are concerned. At the same time, there is evidence that articulatory research can deliver unique insights into vowel variation and change (e.g., Lawson et al., 2019; Strycharczuk & Scobbie, 2016). To extract maximal benefit from articulatory data, we must try to establish and follow good practice. In particular, careful justification is needed for selecting specific articulatory features, especially when these are being used to compare different vowels and/or different groups of speakers. We recommend collecting multiple features in such cases and building a more holistic picture of different parts of the tongue.

**Figure 15.**
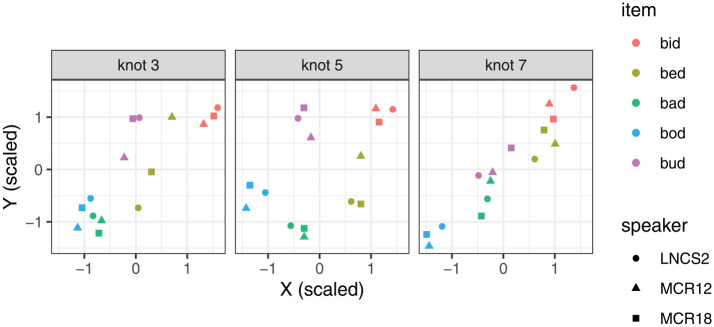
A multi-dimensional representation of scaled X-Y coordinate values for three DLC knots (Knot 3, 5, and 7) for three example speakers.

We propose to extract multiple measurements, consistent with multi-dimensional models of vowel articulation. The error analysis presented in Section 3.2 and the correlation analysis in Section 3.3 bring out the complementary insights delivered by the height of the anterior and posterior points of the dorsum, and by the position of the tongue dorsum vs. tongue root. The key locations relevant here are DLC knots 3, 5, and 7. Figure 15 illustrates a multi-dimensional representation spanning the X-Y coordinates of these three knots. As can be seen in the figure, this approach underscores the involvement of different parts of the tongue in vowel production, and consequently, the differences in the overall vowel geometry, comparing different parts of the tongue. For instance, *bad* is considerably retracted at the tongue root (Knot 3), but not so at the tongue dorsum. The representation also brings out the fact that the *bid*-*bed* contrast is created by the relative fronting of the tongue root, which translates into a height contrast at the anterior part of the dorsum, due to the muscle constriction pushing the tongue volume upward (Gick et al., 2017). Inclusion of the posterior part of the dorsum (Knot 5) captures the location and height of the back vowels, *bod* and *bud* relative to *bad* on the one hand, and *bid* on the other. While the representation involves a degree of redundancy, it serves as a reminder of how different parts of the tongue function together in speech production.

In the context of Figures 14 and 15, it is somewhat curious that the Y coordinate of K7 consistently came out as one of the preferred measures of tongue height, on par with the height of the mid part of the tongue dorsum (K6) and sometimes outperforming maximum tongue height (HP Y). For back vowels, such as *bod* and *bud*, K7 is anterior to the constriction points, and it clearly does not capture the maximum tongue height. On the contrary, these vowels often involve a degree of pre-dorsum concavity, such that there is a dip in height around K7. It is also not strongly correlated with F1 (Figure 11). However, this dimension captures the contrast between back vowels *bud* and *bod*, which are not reliably distinguished by the position of the tongue dorsum (see Figures 12 and 13). This is a possible reason why K7 Y performs well in comparisons focused on articulatory distinctness.

Our recommendation to focus on tongue root and two points on the tongue dorsum is informed by our data, which represent a relatively symmetrical system of dorsal vowels. It may not be universally appropriate because some vowel inventories may involve a different type of articulatory contrast. Some vowels, such as apical vowels, involve the tongue tip as an articulator (Lee-Kim, 2014). To capture the relevant articulatory contrasts for inventories including such vowels, it would be appropriate to include the coordinates of the tongue tip and tongue blade. Similarly, rhotic vowels, may involve contrasts between retroflex (tip-up) and bunched (tip-down) articulations (Delattre & Freeman, 1968; Mielke, 2015; Xing, 2021). Once again, including the tongue tip and /or blade would be important to capture the full range of articulatory contrast, and we expect that measures related to these articulators would surface as important in cross-measure comparisons such as the one we proposed in this study (we also suspect that tongue shape measures would fare relatively well for the retroflex-bunched contrast).

It is also important to consider the extent to which our results are influenced by focusing on lax vowels. In Section 1.3, we noted that lax vowels are less distinct than tense vowels. In consequence, they may present a greater challenge to tasks such as phonemic classification of tongue shape (Sections 2.5.3 and 3.2). To assess this, we carried out the same analysis based on five tense vowels produced by the same speakers: *bead, booed, bird, bard*, and *bored* (note that all our speakers are non-rhotic). The results for tense vowels are available on the OSF. While we cannot discuss them in detail, we note that some differences emerge between the two set of results. Broadly, the same measures emerge as preferred ones for tongue position, but unlike in lax vowels, the position of the tongue root (K3 X) also performs very well. As far as height measures are concerned, we find an overall better consistency in the information captured about vowel contrasts, comparing HP Y, K6 Y, and HV Y. While some small differences emerge, these measures deliver more comparable results than they do for lax vowels. They also show stronger correlations with F1, although interestingly, the correlations are weaker for measures of tongue positions and F2 in tense vowels than in lax vowels. Overall, it seems that some of the issues we have discussed here concerning measures of tongue height are more acute in lax vowels. This, however, only serves to highlight that individual measures work better for some vowels than for others, and therefore, a multi-dimensional analysis is preferable when comparing different vowels, especially if they differ greatly according to tongue position or tenseness/laxness.

Residual issues related to quantifying lingual articulation in vowel production concern normalization and temporal reduction. Raw X-Y Cartesian coordinates are clearly not comparable across speakers, as they vary depending on the location of the probe, and distances expressed using these coordinates are highly sensitive to issues such as vocal tract size. Here, we have relied on *z*-scoring to make the measurements more comparable, but more work is needed to establish whether this approach is appropriate for making comparisons between speakers. As far as temporal reduction is concerned, the acoustic midpoint almost certainly does not systematically correspond to the articulatory vowel target. In principle, kinematic analysis of DLC knot displacement could potentially serve as to obtain a time-varying representation of crucial points on the tongue surface, which could subsequently be reduced to gestural targets based on the systematic changes in the velocity profile. Further work is needed to determine how the movement of the different parts of the tongue relates to a potential more global target that we could interpret at a segmental level.

We expect that future work will refine tongue models, for example, by relating observable displacement to muscle constriction. In the meantime, our results go some way toward validating the DLC model, in that they consistently point to the involvement of the same DLC knots across different speakers, and they show by-vowel differences consistent with known differences about vowel articulation. Such findings could only emerge if the knots identified by the DLC represent functionally equivalent areas of articulation in different speakers. While there is a degree of error associated with DLC, it arguably offers a meaningful approximation of the location of anatomical landmarks. Thus, it offers a whole new way of quantifying articulation to ultrasound studies, making consistent labeling of articulators easily achievable.

## 5 Conclusion

We have considered different ways of reducing tongue contours to two dimensions (meaning a set of two measurements), in the context of quantifying differences between vowels. We focused on physical phonetically identifiable measures inspired by extant models of vowel production. Our evaluation does not clearly support any of the models, nor any specific measure. Instead, it suggests that two-dimensional approaches to quantifying lingual vowel articulation are overly reductive, and that they all generate some systematic error. While our findings do not undermine two-dimensional models, they highlight that such models involve some degree of abstraction, and consequently, they cannot serve as the foundation for extracting reliable articulatory measurements. Instead, we recommend an approach consistent with multi-articulator models that quantify the displacement of the key articulators involved in producing vowel contrasts. In our case, three locations on the tongue contour emerge as the key ones: the tongue root, the posterior part of the tongue dorsum, and the anterior part of the tongue dorsum. However, other locations may also be relevant, depending on the vowel system involved.

## Notes

1. We believe that the negative correlation between the height Knot 5 and F1 arises because the posterior part of the dorsum is at a similar height for high-front and high-back vowels, coinciding with acoustic differences. This is unlike maximum tongue height, which is inter-correlated with position.
